# Nursing students’ readiness towards the ‘new normal’ in clinical practice: a distributed cognition qualitative perspective

**DOI:** 10.1186/s12912-024-01819-x

**Published:** 2024-04-22

**Authors:** Wei How Darryl Ang, Khairul Dzakirin Bin Rusli, Ying Lau, Siew Tiang Lau, Han Shi Jocelyn Chew

**Affiliations:** 1 Alice Lee Centre for Nursing Studies, Yong Loo Lin School of Medicine, National University of Singapore, Singapore, Singapore; 2 The Nethersole School of Nursing, Faculty of Medicine, The Chinese University of Hong Kong, 6-8/F, Esther Lee Building, Shatin, New Territories, Hong Kong China

**Keywords:** Clinical readiness, Resilience, Nursing students, Clinical practice

## Abstract

**Background:**

Clinical practicums are a core component of baccalaureate nursing education. Following the coronavirus pandemic, there have been extensive changes in the workforce environment that may potentially affect nursing students’ experience and readiness for clinical practicums.

**Methods:**

A qualitative study was conducted to explore final-year nursing students’ experiences and readiness for their final clinical practicum before becoming a registered nurse. A purposive sample of 24 final-year baccalaureate nursing students was included in this study. Individual semi-structured interviews were conducted face-to-face via Zoom. The data was analysed using an inductive thematic analysis approach.

**Results:**

Three themes depicting students’ experiences and clinical readiness were elucidated. The themes included: (1) Experiencing multiple concerns, (2) requiring a network of support, and (3) easing the transition to professional practice. Students considered the final clinical practicum as challenging and demanding which evoked numerous concerns.

**Conclusions:**

Considering the stress that final-year nursing students experience, it will be important to devise strategies ranging from personal, relational, and environmental protective factors to enable their successful transition and completion of clinical practice.

**Supplementary Information:**

The online version contains supplementary material available at 10.1186/s12912-024-01819-x.

## Introduction

Globally, clinical practicums form the cornerstone of nursing education. Clinical practicums, where a group of nursing students taught by a clinical instructor, provide students with the opportunity to draw links from the theoretical components in the classroom to the clinical area. As students undergo their clinical practicums, they experience stress from a variety of factors ranging from personal (e.g., lack of opportunities, knowledge gaps), to relational (e.g., relationships with clinical instructors), and environmental reasons (e.g., unfamiliarity with the clinical area) [1, 2]. Contemporarily, a global workforce migration following the coronavirus pandemic has led to massive changes in the healthcare environment [3, 4].

Student nurses have inevitably been affected by these changes in numerous ways. To begin, student nurses are now required to support the healthcare workforce and provide frontline support [5, 6]. In response to the pandemic, students have experienced numerous aberrations in their education journey, with some having to substitute their clinical placements with simulations while others had to study over an online platform [7–9]. For these reasons, students who enrolled during the pandemic (i.e., in 2020 and 2021) and are now in their final year of nursing education may have experienced numerous alterations in their nursing education which may potentially result in poorer readiness for clinical practice.

Adequate preparations for clinical practicums are of utmost importance. Students who are poorly prepared may experience anxiety and stress [10, 11]. Literature has reported that nursing students experienced moderate to high levels of stress during clinical practicums [2, 12]. Understanding stress is important as it can be debilitating and may have a negative influence on nursing students’ clinical performance [13, 14], sense of belonging [15], and physical health [16]. Collectively, the continuous experience of stress and self-perceived lack of competence may expedite nurses’ intent to leave the profession [17, 18].

Several reviews have contributed to an in-depth understanding of nursing students’ experiences in the clinical area [10, 19]. However, students’ experiences and practice readiness may be influenced by the circumstantial differences brought upon by the COVID-19 pandemic that some would refer to as the “new normal” [20]. The differences include extensive changes in the workplace environment such as greater independent learning and students’ learning experiences such as online learning and substituted clinical simulations [20]. Furthermore, considering that these students belong to Generation Z (born between 1995 and 2010), their view on their clinical practicum experiences may differ from earlier findings, as studies have suggested that Generation Z students possess distinct characteristics such as aversion to negative events or having greater influence by the self-esteem drive [21].

Therefore, it is important to understand how these students who are the future generation of nurses experience their final clinical posting and their readiness to transition into registered nurses. The understanding also sheds insights on ways to minimize premature departure by new nurses from the nursing workforce as reported in several studies [22].

Given the potentially wide-ranging factors that may influence students’ perspective of their readiness for clinical practice, a broad and in-depth analysis approach is required. The distributed cognition theory [23] was adopted in this study to achieve a comprehensive analysis approach. The distributed cognition theory [23] is concerned with the organization of cognitive systems that often interact and are dispersed across different dimensions. There are three key dimensions of the theory: physical organization of work, information flow, and artifacts [24]. These dimensions suggest that to understand a perspective or cognition, researchers should go beyond the individual to the interactions between people and resources [25].

The physical organization of the work dimension looks at the structure of the setting or context. For example, new nurses would typically be enrolled in an induction program for them to be aware of the institution’s policies. Therefore, exposure to this program may have an influence on nurses’ cognitive perspective regarding their new work role. The second dimension, information flow, looks at the different aspects of communication and how information flows across time, places, and people. For example, how nurses’ past experiences with people or learning modalities influence their current or future outlook. The artifact dimension looks at how resources are designed to support nurses’ cognitive perspectives such as the availability of support system platforms or a competency checklist.

Although this theory requires a long time to understand and interpret the identified cognitive processes through the three dimensions, it has been adopted in other disciplines such as understanding the perspective of new investment bankers [8], and exploring adapted learning with artificial intelligence [26]. In healthcare, the adoption of the theory is emerging which includes exploring the use of infusion pumps by nurses [27], scoping review of studies using distributed cognition theory on decision-making in acute care [28], and exploring the process of intensive care patient discharge [29]. Thus, the combination of the three dimensions of distributed cognition theory in this study would generate an understanding that is larger than the sum of its parts [25].

## Methods

This qualitative study was reported following the Consolidated Criteria for Reporting Qualitative Research (COREQ) checklist [30] (Supplementary Material 1: Appendix 1). This study was conducted as part of a larger study that evaluated the effects of a clinical readiness program for final-year nursing students. Considering that the researchers have little insight into the topic of interest, a qualitative approach underpinned by the distributed cognition theory was performed to extrapolate the meaning and nuances of these perspectives through the lens of the students [31, 32]. This study sought to address the following research question:

- What are final-year nursing students’ experiences and readiness for their upcoming TTP clinical practicum?

### Setting

This study was conducted between January to May 2023 among final-year undergraduate nursing students at a university in Singapore. The undergraduate program in the university was read by pre-licensure or post-registration nursing students. Pre-licensure nursing students read a three-year bachelor’s program, while post-registration nursing students with a nursing diploma went through a two-year accelerated bachelor’s program. In the final semester of the undergraduate nursing program, all students undergo a 10-week transition to professional practice (TTP) clinical practicum. Unlike clinical practicums in the junior years where students go to the clinical area in groups, TTP is often done in smaller groups, and in certain cases, students are alone during the entire posting. As the TTP is the final clinical practicum, students are expected to perform at a level similar to the registered nurse (e.g., taking case). At the time of this study, the students have not gone for the TTP clinical practicum.

### Sampling strategy and eligibility

A purposive sampling using a maximum variation approach (e.g., age, gender, and ethnicity) was undertaken to recruit eligible participants [33, 34]. Participants were eligible if they were: (1) Above the age of 18 years, (2) in the final year of their undergraduate nursing program, (3) enrolled in the Transition to Professional Practice Experience module, (4) able to comprehend the English language, and (5) have a device that connects to the Internet.

### Data collection

Following ethics approval, an email comprising a research poster was disseminated to all final-year students. Students were provided with a participant information sheet explaining the details of the study. Interested participants indicated their interest via an online survey. Through purposive sampling, selected students were invited to participate in a semi-structured individual interview via Zoom that was audio and video recorded. There were no other non-participants involved in the interviews.

All participants were required to participate in a single interview. The interviews were conducted by one female research assistant with a Bachelor of Arts qualification to ensure consistency. The research assistant does not have a dependent relationship with the participants. Before the start of the interviews, the interviewer built rapport with the participant by sharing her personal goals, role in this study, and aims of this research study. The interviewer received formal qualitative research training and was guided by a doctoral-prepared researcher (AWHD). The interview guide was prepared based on a review of the literature [35, 36], the transactional model of stress and coping [37], and the distributed cognitive theory [23]. Although several pieces of literature on this theory have also suggested direct observation to have a deeper understanding of the topic of interest [38, 39], this was not feasible due to pandemic-induced measures. Instead, probing questions (e.g., examples of an experience) were used in place of direct observations. Two pilot interviews were conducted to assess the comprehensibility and flow of the questions. The feedback from the pilot interviews informed the revision of the interview guide. As there were minimal modifications, the data from the pilot interviews were included in the final analysis. The eventual guide (Supplementary Material 1: Appendix 2) comprised questions relating to participants’ perceptions of their clinical readiness for the upcoming TTP clinical practicum.

The interviews were conducted over two months (February to March 2023). Field notes were recorded during the interview by the interviewer. A total of 24 interviews were conducted with duration ranging from 31 to 57 min (Mean = 40.1 min). Data saturation, which was defined as the point in which no new concepts were identified was achieved at the 22nd participant [40]. Two additional interviews were conducted to confirm data saturation. Interviewees were reimbursed 20 Singapore Dollars for their effort and time in participating in the study. None of the participants dropped out of this study.

### Data analysis

A six-step inductive thematic analysis approach was used by two researchers (AWHD and HSJC) to analyse the data [41]. To begin, one researcher (AWHD) reviewed all the transcripts for completeness and verbatim accuracy. The transcripts were imported into NVivo version 1.7.1 for further analysis and management. Next, the researchers familiarized themselves with the data by reading the transcripts multiple times. The first six transcripts were used to develop the code book. Both researchers compared and reviewed their codes against the aim (i.e., participants’ experience and readiness for the final clinical practicum) and the three dimensions of the distributed cognition theory. A third reviewer (LY) was included to resolve any discrepancies. Consequently, the researchers coded all remaining 18 transcripts using the code books. A total of 155 codes were identified. Codes with similar meanings were arranged into themes and subthemes. A total of three themes were elucidated from participants’ narratives. The developed themes were returned to four participants who were willing to review the data. Following the member checking, there were no changes to themes and subthemes. Finally, the eventual themes were reported narratively and visually through figures. The coding tree can be found in Supplementary Material 1: Appendix 3.

### Rigour

Four criteria [42] were used to establish the rigour of this study’s findings, (1) credibility, (2) transferability, (3) dependability, and (4) confirmability. First, credibility was established based on several strategies throughout the study. During the data collection, there was a prolonged period of engagement with the participants during the interviews and debriefing sessions were held after every interview to discuss the interview process [42]. Finally, during the data analyses, two independent researchers were involved in the data analysis, and member checking was conducted to ensure that the themes were representative of the participants’ narratives [43]. Second, transferability was ensured through a thick depiction of the study context [42]. Third, dependability was established through the maintenance of an audit trail and description of the study processes to enable replicability of the study [42]. Finally, confirmability was ensured by the use of verbatim quotations based on the participants’ narratives and the use of a reflexive journal that reduced any form of researcher-induced biases [42].

### Ethical considerations

This study adhered to the ethical principles based on the Declaration of Helsinki [44]. The recruitment and interviews were conducted by a research assistant who did not have any dependent relationship with the students. Students were provided with information sheets and gave informed consent before their participation. Participants were also informed that their participation was voluntary and they were able to withdraw from the study without any consequences (e.g., no impact on their grade or standing in the program). Helplines such as counselling services were provided to all participants. This study obtained ethical approval from the National University of Singapore’s Institutional Review Board (NUS-IRB-2022-776).

## Results

### Participants’ characteristics

**Fig. 1:**
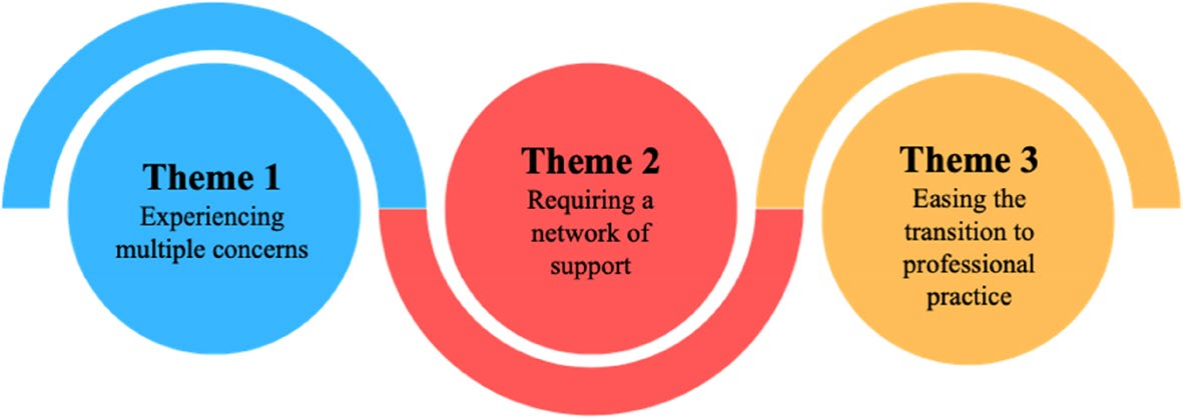
Participants’ experience and readiness for transition to professional practice practicum

A total of 24 final-year baccalaureate nursing students participated in this qualitative study and their sociodemographic characteristics are presented in Table 1. The mean age of the participants is 22.7 years old (Range: 21 to 27 years old). The participants were largely female (75%), ethnic Chinese (67%), and were from the pre-licensure program (79%). The data analysis unveiled three themes about nursing students’ experiences and readiness towards their upcoming TTP clinical practicum: (1) Experiencing multiple concerns, (2) requiring a network of support, and (3) easing the transition to professional practice (Fig. 1).

**Table 1:** Participants’ sociodemographic characteristics

Sociodemographic variables	N (%)
Age^a^	22.7 (1.57)
Gender	
Male	6 (25%)
Female	18 (75%)
Ethnicity	
Bengali	1 (4%)
Chinese	16 (67%)
Filipino	2 (8%)
Malay	1 (4%)
Indian	4 (17%)
Type of program	
Pre-licensure	19 (79%)
Post-registration	5 (21%)

^a^Mean and standard deviation

### Theme 1: experiencing multiple concerns

Considering that the students had to engage in a clinical practicum of greater intensity and longer duration, this theme depicts the participants’ concerns and uncertainties regarding the upcoming TTP clinical practicum. When asked about their concerns, the majority of the students used emotional descriptors such as overwhelming, stamina, and mental resilience to describe their readiness for the upcoming TTP. In one nursing student:

“The feeling of overwhelmedness stems from the fact that this is probably the longest posting … and I wasn’t sure if I would have the stamina and like, mental resilience to carry through the posting”. (P9, Female, Chinese)

Participants attributed it to the heightened intensity, knowledge gaps, and responsibilities that they are expected to demonstrate as one of the reasons for their experienced emotions. The need to perform at a higher competency relating to a registered nurse surfaced in two students’ narratives:

“Usually, the normal attachments, we don’t have to take on so much responsibilities …during TTP, the biggest change is the need to take cases, face patients, communicate with them and their family, and with the team. So, I think that’s a big change”. (P15, Male, Chinese)

“When I’m in clinical, there are many drugs that I am unaware of”. (P8, Female, Chinese)

Collectively, students’ fears may have potentially crippling effects with some experiencing doubts in their abilities. Specifically, the participants experienced concerns about managing the care of their patients, a key requirement for passing the TTP practicum. In one student:

“Am I ready to like take on cases and take care of my patients properly?”. (P19, Female, Chinese)

### Theme 2: requiring a network of support

Aforementioned, TTP is a challenging period and students may experience multifarious vicissitudes in this journey. For this reason, the majority of the students verbalized the need to understand the experiences of former students who have been through TTP and this was often lacking. In one student

“I just felt very lost. I have no one to clarify, there are no seniors to talk to us”. (P12, Female, Chinese)

Speaking to former students has been surfaced to be an important aspect in preparing students for TTP as they are unable to obtain this information from other informal sources of social support. Students verbalized that their existing social networks are insufficient as these individuals have little knowledge about what to expect from TTP. In one student:

“If I am being anxious about my posting, I feel like I don’t have anyone to share about, like if I talk to my family, they wouldn’t understand … All my friends have the same concerns so it’s like you are chatting a lot of concerns but it’s like no one can give me like a pep talk”. (P11, Female, Chinese)

Unlike clinical practicums in the junior years where students go to the clinical area in groups, TTP is often done in smaller groups, and in certain cases, students are alone during the entire posting. Being alone during the clinical practicum can have an impact on students’ mood and their lack of opportunities for social interactions. In one student:

“In TTP, I feel very alone, if I am in the ward, I am the only person there, I am the type who will get very sad … like … break time I eat alone”. (P17, Female, Chinese)

Finally, students highlighted a greater need for locating networks of support as part of building their clinical readiness for TTP. One proposed strategy would be to develop a peer support system through the TTP:

“Like a peer thing … you could have buddy or something and it could be over a period of time”. (P3, Female, Indian)

### Theme 3: easing the transition to professional practice

This final theme depicts participants’ suggestions for easing their transition to professional practice. Specifically, participants alluded to the importance of more knowledge, psychological well-being, and peer support as strategies for enhancing their clinical readiness. First, concerning knowledge, students highlighted the need to gain more understanding of the various interfaces used in the clinical area. They proposed that there could be some form of introductory sessions provided by the healthcare institutions. In one student:

“How to apply your knowledge into practical … teach the students about the different medication systems … it will be good if they can invite the hospital CIs [Referring to Clinical Instructors] over to introduce us to the different hospital policies”. (P7, Female, Chinese)

Due to the extended duration and increased expectations of TTP, students also verbalized the need for more focus on non-cognitive skills. For instance, students highlighted the importance of being resilient during the TTP practicum. In one student:

“For people who have not been to any postings that are like TTP would definitely not be ready for what is to come and what to expect … so, building resilience is very important”. (P14, Female, Chinese)

Nonetheless, some students believe that being consistent would be the key to transit and complete the TTP practicum. In one student:

“At least to me, there is no need to be that afraid, as long as you do your best and you stay consistent and you are on top of your game, it shouldn’t be any different [from other clinical practicums]”. (P24, Male, Filipino)

## Discussion

This study focused on exploring and understanding final-year nursing students’ experiences and readiness toward their clinical practicum in the “new normal”. Through participants’ narratives, the TTP clinical practicum is perceived as a transformative journey where students will transition to becoming registered nurses. This clinical posting is also one fraught with numerous concerns to self-doubts about one’s readiness for TTP. Participants also highlighted a need for identifying a network of social support to enable their transition towards clinical practicum. Finally, students proposed several suggestions to improve their experience for their upcoming clinical practicum.

These narratives generated three themes: (1) Experiencing multiple concerns, (2) requiring a network of support, and (3) easing the transition to professional practice. These themes could be categorized into the three dimensions of distributed cognition. Theme one could be categorized into the “physical organization of work” dimension as the participants highlighted the higher intensity of work and emotions they had while undergoing their final clinical practicum. Theme two could be categorized into the “information flow” dimension as the students highlighted the gaps in having prior understanding and information for their final clinical practicum. The last theme could be categorized into the “artifact” dimension as student highlighted the paucity of systems and resources to support them in their clinical practicum.

### Physical organization of work

Firstly, students viewed the final clinical practicum as one that is overwhelming and extends beyond their usual clinical experiences, a finding that was also reported in previous studies [22, 45]. Considering that the final clinical practicum is often regarded as the bridge towards becoming a registered nurse, it is not uncommon that final-year students are often tasked with higher-order responsibilities such as managing patient care and communicating with numerous stakeholders (e.g., other members of the healthcare team or family members). These similar concerns and challenges were corroborated in several studies [22, 46].

Secondly, this study found that students verbalized concerns relating to TTP from various origins, such as their inability to provide proper care, and knowledge gaps, which were echoed by other final-year nursing students [46, 47]. This was an interesting finding considering that students have undergone several years of clinical practice and are familiar with the roles of a student nurse. It was also surprising that both pre- and post-licensure students experienced similar concerns. This could be possibly due to the lack of recent exposure in the clinical area as the students in the post-licensure program only have a one-month clinical practicum before embarking on the TTP posting. Nevertheless, the concerns that the students experienced may be because the current generation of students grew up during a period of numerous uncertainties and thus they may be more susceptible to experiencing fears [48]. From a theoretical perspective, the transition theory postulates that when an individual experiences a form of change from one state to another, one may experience a state of instability until the end [49]. For this reason, it is plausible that the transition process to the final clinical practicum may have induced certain elements of concern among these students.

### Information flow

Accordingly, due to the increasing complexities of clinical postings, and heightened concerns relating to TTP, students articulated a need for greater social support. The narratives surrounding a need for social support during clinical practicums were also highlighted in other studies [50, 51]. Considering that the current generation of students potentially have underdeveloped social skills due to greater reliance on technology (e.g., using electronic devices), it is not surprising that they expressed such concerns [48, 52]. Interestingly, unlike other studies where faculty plays a bigger role in supporting students [48], this study found that students sought more social support from informal non-clinical sources such as friends and family members. This could be a preference for Generation Z nursing students who prioritized friendships and familial relations [53–55]. According to the social baseline theory [56], proximity plays a role in how an individual gains social support. Therefore, as students are in the clinical area, academic staff may be physically ‘distant’ and are less likely to be an immediate source of support. In addition, this study found that students could gain support for their TTP clinical practicum by learning from other individuals’ experiences. Learning through other individuals’ narratives is one of the preferred by the current students [21].

### Artifacts

Finally, participants proposed a series of multi-level strategies such as personal (e.g., enhancing resilience), relational (e.g., peer support systems), and environmental (e.g., familiarity with the healthcare system) that could improve their readiness before entering TTP. These proposed strategies were similarly outlined in several studies [57, 58]. Regarding the society-to-cell resilience theory [59], an individual may draw from similar multi-level protective factors that assist them in overcoming challenges and adversities. As students contend with the challenges of clinical practicum, it is evident that they will require some form of non-cognitive traits such as resilience to support their journey [51, 60]. Widely reported in the literature is the role of social support in enabling nursing students to be successful in their clinical journey [53, 61, 62]. Social support can offer students with resources through various mechanisms, such as emotional support, or practical [21, 55]. From an environmental perspective, students verbalized the need for more knowledge relating to the healthcare system. Prior research has highlighted the importance of understanding the context of the healthcare system as a key enabler of learning in health sciences [63, 64].

### Limitations

The findings of this study have to be reviewed in the context of several limitations. One, this study was conducted in one nursing department in Singapore and may not be representative of the nursing students. Nonetheless, a purposive sampling approach enabled rich and in-depth experiences from students from different backgrounds. Two, the findings of this study were based only on in-depth qualitative inquiry due to the pandemic restrictions during the data collection stage. Therefore, future studies could adopt an ethnography approach to confirm the findings of the identified themes. Nonetheless, this study seeks examples of experiences from the participants to support their sharing accordingly. Finally, this study was limited to a single time point and the complex experiences of the clinical practicum were not captured. It will be worth conducting a longitudinal study across the entire clinical practicum period to explore students’ readiness.

### Implications for practice and research

Despite the limitations, this study can lend its findings to several implications for practice and research. First, it is now understood that final-year nursing students have several needs ranging from knowledge gaps to psychological well-being as they enter the final clinical practicum. It is therefore important to consider implementing clinical consolidation programs to adequately prepare students for their practicum. Considering that the current generation of students are digital natives, technology-enabled clinical simulations delivered collaboratively with hospital-based educators and preceptors could be used to enhance students’ readiness. For instance, students may be grouped by hospitals and receive contextually relevant information specific to their upcoming clinical practicums. This is a plausible idea as earlier research has shown that the use of such clinical simulations can improve students’ satisfaction and readiness for clinical practice [65, 66].

Second, as final-year clinical practicums are known to be challenging, it will be important to enhance students’ non-cognitive skills such as resilience and teamwork before their posting. Unlike studies that were conducted before the pandemic [22, 46], the present study found that students placed greater emphasis on their mental well-being. Although similar challenges (e.g., complexities of nursing care, lack of social support) were raised, the participants in this study were more directed towards aspects of mental well-being, such as resilience, and loneliness. For these reasons, it will be critical to incorporate psychological well-being programs into the formal nursing curriculum. The current generation of students prefers learning from other individuals’ experiences [67]. Therefore, future psychological well-being interventions should be delivered before the final posting and comprise of alumni’s experiences and their coping strategies. Accordingly, a social support network comprising alumni and current students could be developed to support final-year students’ transition into clinical practicum.

## Conclusion

This study explored the experiences of final-year students and their readiness for clinical practicum. The findings showed that students viewed the TTP as a challenge and fear due to the heightened expectations before becoming a registered nurse. Nevertheless, students proposed the need for more supportive mechanisms to enable their successful completion of the final clinical practicum. The findings of this study can aid in the development of future clinical readiness programs.

### Supplementary Information

**Supplementary Material 1.**

## Data Availability

The data presented in this study are available on request from the corresponding author. The data are not publicly available due to the university’s data sharing policy.
