# Supplementary material for: Nursing students’ readiness towards the ‘new normal’ in clinical practice: a distributed cognition qualitative perspective
Source: BMC Nurs. 2024 Apr 22;23:258. doi: 10.1186/s12912-024-01819-x (PMC11034143; doi:10.1186/s12912-024-01819-x)

**Appendix 1:** COREQ (COnsolidated criteria for REporting Qualitative research) checklist

| **Topic** | **Item No.** | **Guide Questions/Description** | **Reported on**  **Page No.** |
| --- | --- | --- | --- |
| **Domain 1: Research team and reﬂexivity** | | | |
| *Personal characteristics* | | | |
| Interviewer/facilitator | 1 | Which author/s conducted the interview or focus group? | 6 |
| Credentials | 2 | What were the researcher’s credentials? E.g. PhD, MD | 6 |
| Occupation | 3 | What was their occupation at the time of the study? | 6 |
| Gender | 4 | Was the researcher male or female? | 6 |
| Experience and training | 5 | What experience or training did the researcher have? | 6 |
| *Relationship with participants* | | | |
| Relationship established | 6 | Was a relationship established prior to study commencement? | 6 |
| Participant knowledge of  the interviewer | 7 | What did the participants know about the researcher? e.g. personal goals, reasons for doing the research | 6 |
| Interviewer characteristics | 8 | What characteristics were reported about the inter viewer/facilitator? e.g. Bias, assumptions, reasons and interests in the research topic | 6 |
| **Domain 2: Study design** | | | |
| *Theoretical framework* | | | |
| Methodological orientation and Theory | 9 | What methodological orientation was stated to underpin the study? e.g. grounded theory, discourse analysis, ethnography, phenomenology,  content analysis | 4 |
|  | | | |
| Sampling | 10 | How were participants selected? e.g. purposive, convenience, consecutive, snowball | 5 |
| Method of approach | 11 | How were participants approached? e.g. face-to-face, telephone, mail, email | 5 |
| Sample size | 12 | How many participants were in the study? | 6 |
| Non-participation | 13 | How many people refused to participate or dropped out? Reasons? | 6 |
| *Setting* | | | |
| Setting of data collection | 14 | Where was the data collected? e.g. home, clinic, workplace | 6 |
| Presence of non-  participants | 15 | Was anyone else present besides the participants and researchers? | 5 |
| Description of sample | 16 | What are the important characteristics of the sample? e.g. demographic data, date | 8 |
| *Data collection* | | | |
| Interview guide | 17 | Were questions, prompts, guides provided by the authors? Was it pilot tested? | 6 |
| Repeat interviews | 18 | Were repeat inter views carried out? If yes, how many? | 6 |
| Audio/visual recording | 19 | Did the research use audio or visual recording to collect the data? | 6 |
| Field notes | 20 | Were ﬁeld notes made during and/or after the interview or focus group? | 6 |
| Duration | 21 | What was the duration of the inter views or focus group? | 6 |
| Data saturation | 22 | Was data saturation discussed? | 6 |
| Transcripts returned | 23 | Were transcripts returned to participants for comment and/or | 7 |
| **Domain 3: analysis and ﬁndings** | | | |
| *Data analysis* | | | |
| Number of data coders | 24 | How many data coders coded the data? | 7 |
| Description of the coding tree | 25 | Did authors provide a description of the coding tree? | 7 |
| Derivation of themes | 26 | Were themes identiﬁed in advance or derived from the data? | 7 |
| Software | 27 | What software, if applicable, was used to manage the data? | 7 |
| Participant checking | 28 | Did participants provide feedback on the ﬁndings? | 7 |
| *Reporting* | | | |
| Quotations presented | 29 | Were participant quotations presented to illustrate the themes/ﬁndings?  Was each quotation identiﬁed? e.g. participant number | 9-11 |
| Data and ﬁndings consistent | 30 | Was there consistency between the data presented and the ﬁndings? | 9-11 |
| Clarity of major themes | 31 | Were major themes clearly presented in the ﬁndings? | 9-11 |
| Clarity of minor themes | 32 | Is there a description of diverse cases or discussion of minor themes? | 9-11 |

**Appendix 2:** Interview guide

| **Questions** | |
| --- | --- |
| Experience of TTP practicum | 1. How did you feel about the upcoming TTP practicum? 2. What are some of your concerns about the upcoming TTP practicum? 3. What are the factors that can support your clinical readiness for TTP practicum? 4. What are the factors that can negatively affect your clinical readiness for TTP practicum? |
| Overall experience of PRE-TTP program | 1. What was your experience in using the PRE-TTP program? 2. Was the program useful?    1. If yes, why? If not, why not? 3. Did you encounter any issues with the training platform? 4. How did you feel about the online workshop? 5. How did you feel about the practical exercises? 6. Was the duration and length of each video useful? 7. How did you feel about the duration of the online program? 8. Was one week an appropriate time? What would be a better time frame? |
| Contents of PRE-TTP program | 1. Let us understand more about your thoughts regarding the contents of the PRE-TTP program, how was your experience? 2. May you share what you perceive was good? 3. May you share what you perceive was bad? 4. How may we improve the program? 5. Were there any particular videos you found useful? 6. Were there any videos that you did not find useful? 7. Were there any components that we did not cover in the program? 8. Is there anything else you would like to share that I have not covered? |
| Outcomes | 1. How did the PRE-TTP program help or not help you with:    1. Psychological outcome (depression, anxiety, and stress)    2. Resilience    3. Social emotional skill    4. Nursing practice readiness    5. Confidence |

**Appendix 3:** Coding tree


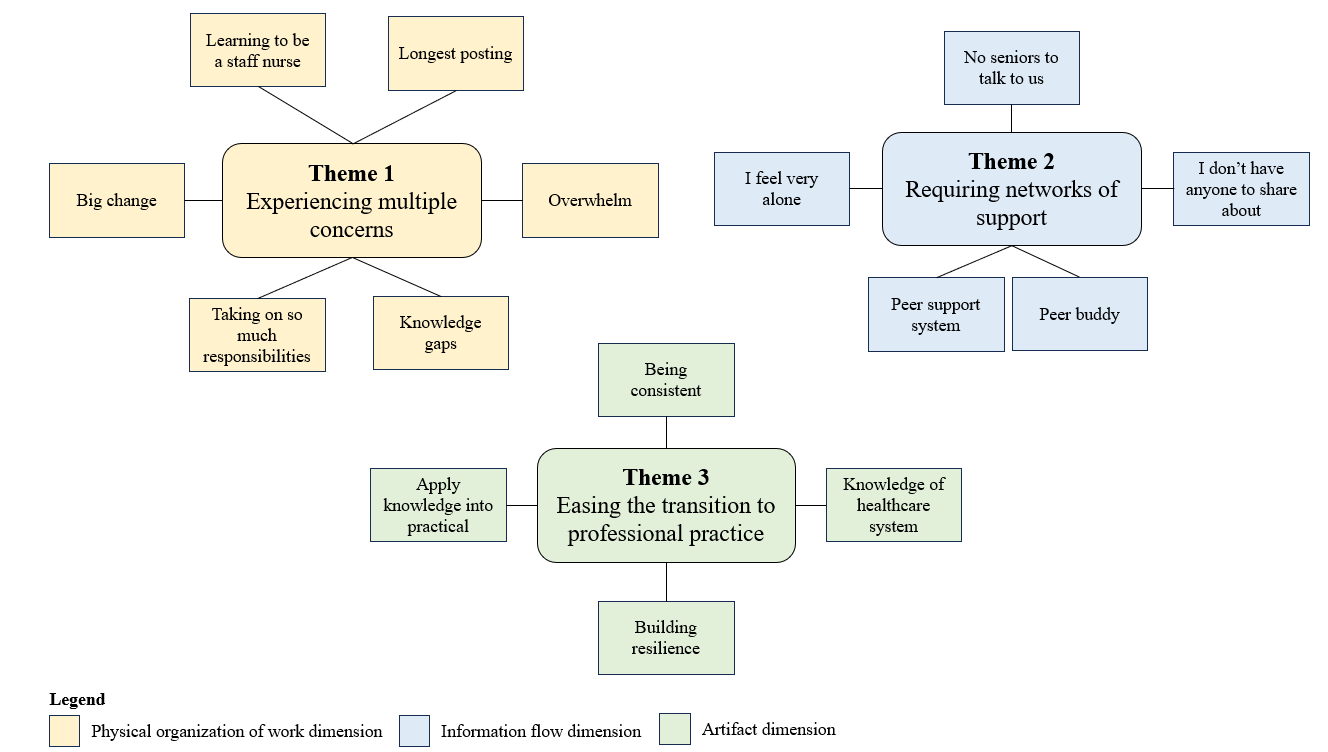

Supplement: Supplementary file 1 — Supplementary Material 1. [file 12912_2024_1819_MOESM1_ESM.docx]
